# Thyroid Dysfunction in Patients with Metabolic Syndrome: A Cross-Sectional, Epidemiological, Pan-India Study

**DOI:** 10.1155/2018/2930251

**Published:** 2018-12-25

**Authors:** Vaishali Deshmukh, Faraz Farishta, Milind Bhole

**Affiliations:** ^1^Shree Hospital, Pune, India; ^2^FS Endocrine & Diabetic Centre, Hyderabad, India; ^3^Abbott India Ltd, Mumbai, India

## Abstract

**Background:**

This study was conducted to assess the prevalence and clinical and epidemiological factors of thyroid dysfunction (TD) in Indian patients diagnosed with metabolic syndrome (MetS).

**Methods:**

In this cross-sectional study, 432 adults with an established diagnosis of MetS were enrolled across ten centers in India. Anthropometric measurements and vital signs were noted. Blood samples were tested for hemogram, coagulogram, lipid profile, and thyroid function. Fasting plasma glucose (FPG) and fasting plasma insulin were used for the calculation of homeostasis model assessment-estimated insulin resistance (HOMA-IR). Overt hypothyroidism was defined as thyroid-stimulating hormone (TSH) > 4.50 *μ*IU/mL with free thyroxine (FT4) < 0.8 ng/dL and free triiodothyronine (FT3) < 1.4 pg/mL; subclinical hypothyroidism as TSH > 4.50 *μ*IU/mL with FT4 = 0.8-1.8 ng/dL and FT3 = 1.4-4.4 pg/mL; overt hyperthyroidism as TSH < 0.45 *μ*IU/mL with FT4 > 1.8 ng/dL and FT3 > 4.4 pg/mL; and subclinical hyperthyroidism as TSH < 0.45 *μ*IU/mL with FT4 = 0.8-1.8 ng/dL and FT3 = 1.4-4.4 pg/mL.

**Results:**

About 121 out of 432 patients (28%) were diagnosed with TD (mean age ± SD: 47.9 ± 10.96 years), with women predominance (75% versus 25%). Most patients were in the >45 years of age group (men: 63%; women: 59%). TD was associated with high waist circumference (99.17%), reduced high-density lipoprotein-C (87.60%), raised HOMA-IR (86.78%), systolic blood pressure (77.69%), diastolic blood pressure (59.50%), fasting glucose (58.68%), and triglycerides (33.06%). Overt hypothyroidism was reported in 17.59% (*N* = 76) of patients. Subclinical hypothyroidism, overt hypothyroidism, and subclinical hyperthyroidism were reported in 8.10%, 1.60%, and 0.70% patients with newly occurred TD, respectively. No case of overt hyperthyroidism was present in these patients.

**Conclusion:**

Hypothyroidism was the most common TD in Indian patients with MetS. A large proportion of TD cases diagnosed during the study highlight the need for vigilant thyroid screening in patients with MetS in a real-life setting.

## 1. Introduction

Metabolic syndrome (MetS) is a combination of risk factors such as hypertension, atherogenic dyslipidemia, hyperglycemia, truncal (central) obesity, and prothrombotic and proinflammatory conditions, which could increase the risk of cardiovascular illness, diabetes, and death. According to an estimate by the International Diabetes Foundation, nearly one-fourth of the world's population has MetS [1]. The prevalence rates vary greatly depending upon the definition of MetS, ethnicity, age, population, etc. Recently, a rapid increase in its prevalence has been noted in India due to socioeconomic transitions to increasing affluence, urbanization, mechanization, and urban migration [2]. About one-third of the urban population in large Indian cities has MetS [3] with the overall prevalence varying between 11% and 56% [4].

Thyroid diseases are among the most prevalent endocrine disorders worldwide. Based on the estimation from various studies, it has been projected that about 42 million people in India suffer from thyroid diseases [5]. MetS is closely associated with thyroid dysfunction (TD) due to the impact of thyroid hormones on lipid metabolism, glucose, blood pressure, and cardiovascular dysfunction [6]. Functional changes in the thyroid gland might have an association with MetS and its related components including obesity, insulin resistance (IR), lipid and glucose metabolism abnormalities, raised blood pressure, and cardiovascular dysfunction. MetS and TD are both characterized by a cluster of common abnormalities such as abdominal obesity, hyperglycemia, hypertension, reduced high-density lipoprotein cholesterol (HDL-C), and elevated triglycerides (TG). Moreover, IR, identified as a basic mechanism for MetS, also plays a role in hypothyroidism [7]. The occurrence of both the conditions may be compounded to increase the risk for cardiovascular diseases (CVDs).

Several studies have shown a correlation between thyroid function and the indices of MetS [8–11]. Our study assessed the prevalence of TD in Indian patients with MetS to add to the epidemiological data. It was also aimed at exploring the clinical profile and associated risk factors of TD in MetS patients.

## 2. Materials and Methods

### 2.1. Study Settings and Participants

In this multicenter cross-sectional study, adult patients with an established diagnosis of MetS were enrolled across ten sites in India between October 2016 and January 2017. The study protocol was approved by local independent ethics committees. The study was conducted in accordance with the principles of the Declaration of Helsinki, International Council on Harmonization Good Clinical Practice (ICH GCP) guidelines, and Indian regulatory guidelines (Indian Council of Medical Research and Indian GCP guidelines). The study was registered on a publicly accessible database of clinical trials in India with registration number CTRI/2016/09/007270.

Patients aged 18 to 65 years, with an established diagnosis of MetS based on the National Cholesterol Education Program (NCEP) Adult Treatment Panel (ATP) III criteria (with modified waist), with or without known TD, were invited to participate in the study during their routine clinical visit to the endocrinologists, gastroenterologists, and/or hepatologists. Pregnant patients or patients with a history of jejunoileal bypass, biliopancreatic diversion, extensive small bowel resection, total parenteral nutrition, any forms of chronic liver disease, hepatocellular carcinoma, patients on weight loss therapies or steatogenic drugs, and known HIV-positive cases were excluded from the study. At the screening visit, the following data were collected in the case report forms: demographics, anthropometric measurements, significant medical (including FPG, serum TG, and HDL-C values from patients' medical records) and surgical history, family history, lifestyle parameters, history of consumption of any obesogenic medicines, vital signs, and details of physical examination.

After obtaining an informed signed consent to participate in the study, the eligible patients from the screening visit were requested to visit the clinic after an overnight fast within 3-10 days of consenting. At this visit (visit 1), abdominal ultrasound examination (USG) was performed in patients who did not consume alcohol or consumed less than 20 g of alcohol per day and had not received corticosteroids, amiodarone, or tamoxifen. Blood samples were collected for assessment of hemogram, coagulogram (activated partial thromboplastin time, thrombin time, and prothrombin time), plasma insulin, plasma glucose, lipid profile (TG, total cholesterol (TC), HDL-C, and low-density lipoprotein cholesterol (LDL-C)), and thyroid function (free triiodothyronine (FT3), free thyroxine (FT4), and thyroid-stimulating hormone (TSH)). The fasting plasma glucose and plasma insulin were used for the calculation of homeostatic model assessment-established IR (HOMA-IR). The patients were followed up for a mean of one year to check for new diagnoses of TD.

### 2.2. Study Endpoints

The primary endpoint was the prevalence of TD among patients with MetS. Other endpoints included the percentage of patients with hyperthyroidism, subclinical hyperthyroidism, hypothyroidism, and subclinical hypothyroidism and percentage of patients with TD with respect to individual components of MetS (waist circumference, TG, HDL-C, SBP, DBP, and fasting glucose) and IR (HOMA − IR > 1.64).

## 3. Definitions

### 3.1. Body Mass Index (BMI)

The BMI cutoff limits for overweight and obesity were as follows: overweight (BMI ≥ 23 and <25), class I obesity (BMI ≥ 25 and <30), and class II obesity (BMI ≥ 30) [12].

### 3.2. Insulin Resistance

Insulin resistance was assessed using the HOMA score. The cutoff for IR was HOMA-IR score > 1.64 [13].

### 3.3. Metabolic Syndrome

The MetS was diagnosed based on the NCEP-ATP III criteria. The MetS was present, if ≥3 risk factors of the following five criteria were met: abdominal obesity (waist circumference: men: >102 cm (>40 in), women: >88 cm (>35 in)); TG (≥150 mg/dL); HDL-C (men: <40 mg/dL, women: <50 mg/dL); blood pressure (≥130/≥85 mmHg); and fasting glucose (≥110 mg/dL) [14].

### 3.4. Thyroid Dysfunction

TD was graded as described in Table 1 [15].

### 3.5. Statistical Analysis

#### 3.5.1. Sample Size

Assuming that the prevalence rate of hypothyroidism in patients with MetS was approximately 26% [10], the sample size required to construct a 95% confidence interval (CI) around the estimated prevalence rate with 5% margin of error on both sides was calculated to be around 296. Since the prevalence of TD was studied on the same set of patients with MetS, based on the above sample size estimates, a sample size of approximately 400 patients was considered in this study.

#### 3.5.2. Statistical Methods

All statistical analyses were performed using SAS® version 9.2 (SAS Institute Inc., USA). The prevalence of TD in MetS patients was calculated as a number and percentage with 95% CI.

## 4. Results

### 4.1. Demographics and Baseline Characteristics

In this study, we had enrolled 432 patients with MetS. The baseline demographic characteristics of these patients are shown in Table 2. Of all the enrolled patients, 121 (28%; 95% CI: 23.83-32.32) were diagnosed with TD (mean age (SD): 47.9 (10.96) years; mean BMI: 30 ± 4.94 kg/m^2^), with a higher prevalence among women compared to men (91 (75%) vs. 30 (25%)). Most patients (men: 63% vs. 37%; women: 59% vs. 41%) were in the >45 years of age group.

### 4.2. Categorization of TD Patients Based on Thyroid Classification

Of the 432 MetS patients, overt hypothyroidism was reported in 76 (17.59%) patients and overt hyperthyroidism in 7 (1.62%) patients (Table 3). Subclinical hypothyroidism, overt hypothyroidism, and subclinical hyperthyroidism were reported in 8.10%, 1.60%, and 0.70% patients with newly diagnosed TD, respectively. No case of overt hyperthyroidism was detected in these patients.

### 4.3. Percentage of TD Patients for Each of the MetS Components

**Figure 1:**
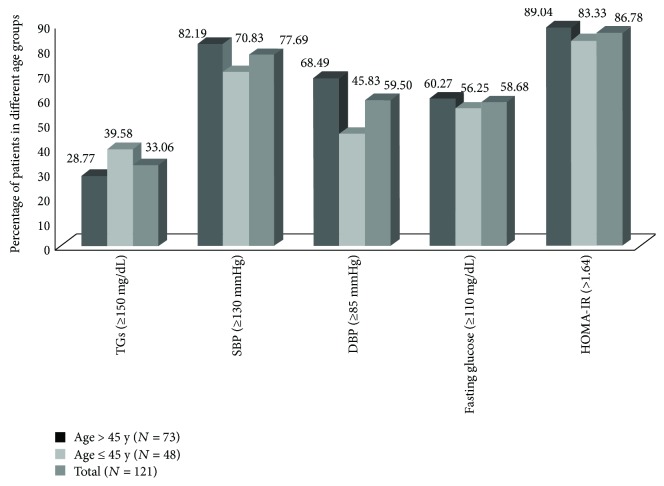
Percentage of TD patients in different age groups with MetS components and insulin resistance.

The most common MetS components associated with TD were high waist circumference (121 (99.17%); women (>80 cm): 75.21%; men (>90 cm): 23.97%), reduced HDL-C (87.60%), systolic blood pressure (94 (77.69%)), diastolic blood pressure (94 (59.50%)), fasting glucose (71 (58.68%)), TG (40 (33.06%)), and elevated HOMA-IR (>1.64) (105 (86.78%)). All the MetS components except TG were more prevalent in patients with >45 years age group (Figure 1).

## 5. Discussion

Thyroid hormones play an essential role in regulating energy balance and metabolism of glucose and lipids, thereby affecting the MetS parameters, including HDL-C, TG, blood pressure, and plasma glucose. Hypothyroidism is found to be associated with obesity, dyslipidemia, and increased risk of atherogenic CVD [16]. In subjects with hypothyroidism, IR is suggested as the possible underlying pathophysiological basis for glucose intolerance when present [17].

Oxidative stress, chronic inflammation, and angiogenesis are believed to enhance the pathogenesis of MetS [18]. The important components of MetS, such as hyperglycemia and inflammation, upsurge the production of reactive oxygen species (ROS) resulting in increased oxidative stress with overactivation of nicotinamide adenine dinucleotide phosphate (NADPH) oxidase [19, 20]. The main ROS is the superoxide anion, produced by NADPH oxidase [19]. Hypermetabolic state in hyperthyroidism may accelerate free radical production in mitochondria and induce changes in the antioxidant defense system. In hypothyroidism, associated oxidative stress is the consequence of reduced capacity of the antioxidative defense.

In our study, 28% of the patients with MetS were diagnosed with TD. This is in agreement with the results of other studies from India [10, 21, 22]. The predominance of hypothyroidism (overt and subclinical: 25.7%) suggests that MetS could also be a consequence or sequel of various grades of hypothyroidism during the natural course of the disease. The new occurrences of TD were mostly as subclinical hypothyroidism (8.10%) followed by overt hypothyroidism (1.60%). Other studies have also shown similar results in which a predominance of subclinical hypothyroidism (14.6% to 53%) was reported followed by overt hypothyroidism (3.5% to 7.4%) [10, 23–26]. The lesser number of subclinical hypothyroidism cases (8.10%) reported in our study could be attributed to the high enrolment of patients with known cases of hypothyroidism (16%), who were already on levothyroxine therapy. The overall rate of hypothyroidism at baseline (16%) plus new overt hypothyroidism (1.60%) and new subclinical hypothyroidism (8.10%) was calculated as 25.70%. In agreement with our findings, a similar prevalence of hypothyroidism was reported in MetS population in other Indian studies as well, viz., Kota et al. (26%) [10] and Shantha et al. (29.3%) [23].

In our study, women with MetS had a higher incidence of TD in comparison to men (21.06% vs. 6.94%). This corroborates reports from other studies where women outnumbered men in terms of prevalence of TD in MetS [27–29]. Both men and women in the higher age group (>45 years of age) had a higher incidence of TD (men: 63% vs. 37%; women: 59% vs. 41%). Our results were in agreement with other reports where there was a tendency of increasing TD with aging across both genders [29, 30]. Thus, age and gender could represent significant risk factors for TD in MetS patients, prompting a detailed clinical and laboratory evaluation in these groups.

In this study, the MetS components observed in patients diagnosed with TD were high waist circumference, reduced HDL-C, raised HOMA-IR, systolic blood pressure, diastolic blood pressure, fasting glucose, and TG. A higher proportion of females had waist circumference above the cutoff (>80 cm) as compared to men (>90 cm; 75.21% vs. 23.97%). Though the other studies have also reported an association between TD and components of MetS, but it is still debatable. A Nigerian study reported MetS to be significantly associated with higher FT4 [11]. Kota et al. found the significant association between subclinical hypothyroidism and MetS with the relationship between TSH levels and TC, TG, LDL, and HDL-C levels among Indian patients [10]. However, a study from Turkey reported no association between TSH and the various components of MetS, though the MetS components other than HDL-C were correlated with total T3, FT4, and FT3/FT4 ratio [31].

It should be noted that while exploring the relationship between TD and components of MetS, most studies have focused on the subclinical hypothyroidism. Further, it should be noted that the pattern of TD in MetS and its relationship with components may vary upon geographic locale, age, gender, diet, and genetics, and environmental factors [23, 30, 32].

There are a few limitations of the present study. First, as this was a cross-sectional study, a cause and effect relationship could not be determined. Second, the grades of hypothyroidism in the subjects who were already on levothyroxine supplement could not be assessed due to lack of adequate previous data. Moreover, a cohort study is needed to evaluate the deleterious effect of TD on metabolic functions. Third, the iodine nutrition status or thyroid autoimmunity was not assessed in the study. Fourth, the impact of age, gender, and body weight on thyroid functions was not assessed in the study.

To conclude, the prevalence of TD in patients with MetS was high, indicating a possible interplay between thyroid status and MetS. Hypothyroidism was the most common TD in Indian patients with MetS. The data generated from the present study will aid in establishing a correlation between TD and MetS in Indian patients. This data will have prognostic importance for practitioners in their routine clinical practice to develop strategies for better management of their patients of TD with associated MetS. This early diagnosis of TD in MetS would help in modifying the disease course by early interventions with appropriate lifestyle modification regimens, as applicable. However, future large sample-sized prospective studies are warranted which could evaluate the impact of TD management in terms of reduction in MetS and its related components.

## Figures and Tables

**Table 1:** Definitions for different grades of thyroid dysfunction.

Parameters	Hyperthyroidism		Hypothyroidism	
	Overt	Subclinical	Overt	Subclinical
TSH (*μ*IU/mL)	<0.45	<0.45	>4.50	>4.50
FT4 (ng/dL)	>1.8	0.8-1.8	<0.8	0.8-1.8
FT3 (pg/mL)	>4.4	1.4-4.4	<1.4	1.4-4.4

TSH: thyroid-stimulating hormone; FT4: free thyroxine; FT3: free triiodothyronine.

**Table 2:** Demographics and baseline characteristics of 432 patients with metabolic syndrome.

Parameter	Age ≤ 45 y (*N* = 177)	Age > 45 y (*N* = 255)	Total (*N* = 432)
Age in years			
Mean (SD)	36.7 (6.08)	55.6 (5.52)	47.9 (10.96)
Range	21.0-45.0	46.0-65.0	21.0-65.0
Gender			
Women, *N* (%)	104 (58.76%)	149 (58.43%)	253 (58.56%)
Men, *N* (%)	73 (41.24%)	106 (41.57%)	179 (41.44%)
Height in cm, mean (SD)	163.0 (9.02)	160.8 (8.44)	161.7 (8.74)
Weight in kg, mean (SD)	79.4 (13.13)	75.8 (12.29)	77.3 (12.75)
Waist circumference in cm, mean (SD)	98.5 (9.41)	98.7 (9.92)	98.6 (9.70)
Hip circumference in cm, mean (SD)	106.3 (11.07)	104.7 (11.14)	105.4 (11.13)

SD: standard deviation.

**Table 3:** Percentage prevalence of different grades of thyroid dysfunction.

Classification of TD	MetS patients (*N* = 432) *N* (%)
Hypothyroidism^∗^	69 (16.00)
New overt hypothyroidism	7 (1.60)
New subclinical hypothyroidism	35 (8.10)
Hyperthyroidism^∗^	7 (1.60)
New overt hyperthyroidism	0
New subclinical hyperthyroidism	3 (0.70)
Total number of TD patients	121 (28.00)

TD: thyroid dysfunction; MetS: metabolic syndrome. ^∗^Known cases of thyroid dysfunction.

## Data Availability

The data used to support the findings of this study are included within the article.
